# Comparative Analysis of the Exo-Erythrocytic Development of Five Lineages of *Haemoproteus majoris*, a Common Haemosporidian Parasite of European Passeriform Birds

**DOI:** 10.3390/pathogens12070898

**Published:** 2023-06-30

**Authors:** Mélanie Duc, Tanja Himmel, Josef Harl, Tatjana Iezhova, Nora Nedorost, Julia Matt, Mikas Ilgūnas, Herbert Weissenböck, Gediminas Valkiūnas

**Affiliations:** 1 Nature Research Centre, Akademijos 2, 08412 Vilnius, Lithuania; tatjana.jezova@gamtc.lt (T.I.); mikas.ilgunas@gamtc.lt (M.I.); gediminas.valkiunas@gamtc.lt (G.V.); 2 Department for Pathobiology, Institute of Pathology, University of Veterinary Medicine Vienna, Veterinärplatz 1, 1210 Vienna, Austria; tanja.himmel@vetmeduni.ac.at (T.H.); josef.harl@vetmeduni.ac.at (J.H.); nora.nedorost@vetmeduni.ac.at (N.N.); julia.matt@vetmeduni.ac.at (J.M.); herbert.weissenboeck@vetmeduni.ac.at (H.W.)

**Keywords:** *Haemoproteus*, birds, megalomeronts, cytochrome *b*, histology, chromogenic in situ hybridization

## Abstract

*Haemoproteus* parasites (Apicomplexa, Haemosporida) are widespread pathogens of birds, with a rich genetic (about 1900 lineages) and morphospecies (178 species) diversity. Nonetheless, their life cycles are poorly understood. The exo-erythrocytic stages of three *Haemoproteus majoris* (widespread generalist parasite) lineages have been previously reported, each in a different bird species. We aimed to further study and compare the development of five *H. majoris* lineages—hCCF5, hCWT4, hPARUS1, hPHSIB1, and hWW2—in a wider selection of natural avian hosts. A total of 42 individuals belonging to 14 bird species were sampled. Morphospecies and parasitemia were determined by microscopy of blood films, lineages by DNA-barcoding a 478 bp section of the cytochrome *b* gene, and exo-erythrocytic stages by histology and chromogenic in situ hybridization. The lineage hCWT4 was morphologically characterized as *H. majoris* for the first time. All lineage infections exclusively featured megalomeronts. The exo-erythrocytic stages found in all examined bird species were similar, particularly for the lineages hCCF5, hPARUS1, and hPHSIB1. Megalomeronts of the lineages hWW2 and hCWT4 were more similar to each other than to the former three lineages. The kidneys and gizzard were most often affected, followed by lungs and intestines; the site of development showed variation depending on the lineage.

## 1. Introduction

*Haemoproteus* species (Apicomplexa, Haemosporida, Haemoproteidae) are parasites of birds and found on all continents except Antarctica [1,2]. These pathogens multiply asexually and produce gametocytes in their avian hosts before being transmitted to their dipteran vectors (biting midges, Ceratopogonidae), in which the fusion of the gametes and the development of sporozoites occur [1,3].

Anemia is an acknowledged symptom caused by *Haemoproteus* parasites, with the gametocytes developing in the erythrocytes [4,5]. However, the exo-erythrocytic development, which occurs in different organs, was initially assessed to be less harmful for their avian hosts compared with that of haemosporidian parasites of the genera *Plasmodium* and *Leucocytozoon* [6], and research on *Haemoproteus* tissue stages remained scarce. Recent studies reported exo-erythrocytic stages of several *Haemoproteus* species [7,8,9,10,11,12,13,14,15], not only increasing and changing traditional knowledge of these parasites but also pointing out how little is still known about their development in avian hosts.

Most reports of exo-erythrocytic stages are either from hosts found naturally dead [7,8,10,11,15] or from individuals that have been purposely targeted for their parasite infection and euthanized [9,12,13,14]. In several cases, no exo-erythrocytic stages of *Haemoproteus* spp. were found in histological slides of birds confirmed positive by blood film microscopy and/or DNA barcoding, despite extensive searches [11,13,14,15]. 

Haemosporidian parasites are investigated and characterized genetically by the amplification and sequencing of a 478 bp DNA-barcode region of the mitochondrial cytochrome *b* (*cyt b*) gene [16]. Morphospecies are usually described using the morphological characteristics of gametocytes and their host cells in blood films from infected birds [1,17,18]. However, not all described species have their molecular characterization, and not all lineages are linked to a species yet. In some cases, several lineages can be linked to the same morphospecies, e.g., *H. majoris* [9,19] and *H. tartakovskyi* [20] (MalAvi database, Lund University, Lund, Sweden, http://130.235.244.92/Malavi/ (accessed on 25 June 2023). [16]).

*Haemoproteus majoris* is a species of interest among the *Haemoproteus* parasites, with several lineages (hCCF5, hPARUS1, hPHSIB1, hPHYBOR04, and hWW2) (MalAvi database [16]; [9,19]) reported in 54 species of birds in Asia, Europe, Africa, and North America (MalAvi database [16]). A sixth lineage, hCWT4, has been associated with this species [21], but it has never been formally described morphologically as *H. majoris*. This species has been studied for its specificity (it is a generalist parasite [21]), its vectors (*Culicoides impunctatus* is a competent vector for the lineages hPARUS1 and hPHYBOR04 [9,22]), and its exo-erythrocytic development (parasites of the lineages hPARUS1, hPHYBOR04 [9], and hPHSIB1 [12] developed into megalomeronts). The exo-erythrocytic stages of *H. majoris* were reported in three different bird species (*Parus majoris*, *Turdus pilaris* [9], and *Phylloscopus sibilatrix* [12]), and all found tissue stages were megalomeronts of similar morphologies, which were predominantly seen in the kidneys. However, it is unknown whether all *H. majoris* lineages develop similarly in different avian hosts. Further comparative studies are needed to answer this question, which is an important issue in understanding the mechanisms of exo-erythrocytic development during avian haemoproteosis. Due to the large diversity of lineages and the occurrence in a large variety of different bird hosts, *H. majoris* is an excellent model organism for such a comparative study.

We aimed to clarify the identity of hCWT4 as *H. majoris* and to investigate and provide a comparative analysis of the exo-erythrocytic stages of different *H. majoris* lineages (hCCF5, hCWT4, hPARUS1, hPHSIB1, and hWW2) in different host species and in different seasons. Patterns of development within this parasite species were investigated in regard to the exo-erythrocytic stages’ morphology, distribution, and localization.

## 2. Materials and Methods

### 2.1. Sampling

Birds caught at the Ornithological Station, Ventes Ragas, Lithuania (55°20′38.93″ N, 21°11′34.05″ E), were sampled and investigated for haemosporidian parasites beginning in May 2016 to 2019, 2021, and 2022 (spring) and in September 2020 (autumn). Birds were additionally caught using mist nests in the Labanoras Forest, Lithuania (55°12′25.77″ N, 25°56′26.47″ E), in June 2018 and 2019 (spring) and July 2020 (summer). Blood was collected by puncturing the branchial vein of the bird. A drop of blood was immediately used to prepare at least three blood films, which were air-dried and fixed with absolute methanol [1]. Blood collected in a heparinized capillary was transferred into a SET buffer (0.05 M Tris, 0.15 M NaCl, 0.5 M EDTA, pH = 8.0) [23] tube for molecular analysis and stored at +4 °C in the field and at −20 °C in the laboratory.

One blood film per individual bird was immediately stained for 15 min with a 30% Giemsa solution and examined for approximately 10 min with an Olympus CX26 microscope (Olympus, Tokyo, Japan) to determine potential infections in captured birds in the field. This procedure enabled the selection of targeted infected birds and the release of all other individuals at the study sites. The remaining blood films were stained with a 10% Giemsa solution for 1 h. Birds infected with *H. majoris*, as observed by microscopic examination, were euthanized, and internal organs were collected; brain, heart, lungs, liver, kidneys, spleen, and skeletal muscles were collected from birds of all years, and trachea, esophagus, gizzard, intestine, pancreas, and reproductive organs were additionally collected from birds collected in 2019 to 2022. The organs were fixed in 10% formalin and later embedded in paraffin wax.

In July 2021, one black redstart (*Phoenicurus ochruros*) found dead in Lithuania (54°52′58.4″ N 25°25′38.6″ E) with a skull fracture was frozen and brought to the P. B. Šivickis Laboratory of Parasitology, Nature Research Centre, Lithuania. In October 2021, one long-tailed tit (*Aegithalos caudatus*) found dead at the Ventes Ragas Ornithological Station, was frozen until dissection. Both birds were dissected, the heart was pressed to a glass slide to prepare a blood film, little pieces of organs were collected in 96% EtOH and stored at +4 °C, and the organs were fixed in formalin for 24 h, washed in distilled water for 1 h, transferred to 70% EtOH, and processed as the other samples. 

### 2.2. Blood Film Microscopy

Haemosporidian parasite species were determined by screening the blood films with an Olympus BX61 microscope (Olympus, Tokyo, Japan) at ×1000 magnification [1]. Parasitemia intensity was calculated as the number of infected erythrocytes per 1000 erythrocytes or per 10,000 erythrocytes in case of low infection and expressed as percentage [24].

Images of the parasites were taken using the same microscope equipped with an Olympus DP70 digital camera and AnalySIS FIVE software (Olympus, Tokyo, Japan). The software Adobe InDesign CS6 (Adobe, https://www.adobe.com/products/indesign.html (accessed on 25 June 2023)) was used to prepare the gametocytes figure.

### 2.3. Histological Analysis

Histological sections of 2–3 μm were cut with a microtome, mounted on glass slides, stained using haematoxylin and eosin (H&E), and investigated for exo-erythrocytic stages using ×100, ×200, and ×400 magnifications with an Olympus BX41 or BX51 microscope (Olympus, Japan). 

Chromogenic in situ hybridization (CISH) was applied on additional sections, at least one per individual, following the original protocol [8,25]. A genus-specific oligonucleotide probe (Haemo18*S*), which targets the *18S* ribosomal RNA of *Haemoproteus* parasites, was used to confirm the generic origin of the observed tissue stages [8].

Pictures of the tissue stages were taken using the camera DP12 with the software DP-SOFT or the camera UC90 with the software cellSens (Olympus, Tokyo, Japan), respectively. The software CorelDraw 2019 (RRID:SCR_014235, https://www.coreldraw.com/en/) was used to prepare the figures. (accessed on 9 November 2022)

For all bird individuals single-infected with *H. majoris* hPARUS1, the number of sections investigated and the number of sections positive for exo-erythrocytic stages were counted and reported. The number of very young megalomeronts and other megalomeronts (referring to growing, mature, and ruptured megalomeronts) were also counted and reported. It is to be noted that very young megalomeronts can be found on up to three sequential sections of organs, but most often, one very young megalomeront was observed in only one section. Other, bigger megalomeronts are most often observed on several sequential sections. However, not all sections investigated were sequential. Therefore, the detection of megalomeronts is biased greatly for big megalomeronts compared with very young megalomeronts. Due to the fact that the actual shape (oval, round, long, or slender) of megalomeronts is difficult to predict, the choice was made to not try to distinguish the real number of the bigger megalomeronts.

### 2.4. Molecular Analysis

DNA extractions were done using the SET-buffer-stored blood following the ammonium acetate protocol [26] or the DNeasy Blood & Tissue Kit (QIAGEN, Venlo, Netherlands). The extracted DNA was used as template in a nested PCR following the original protocols [23,27] with the outer primer pair HAEMNFI/HAEMNR3 and inner primer pairs HAEMF/HAEMR2 for detection of *Haemoproteus* and *Plasmodium* parasites and HAEMFL/HAEMR2L for *Leucocytozoon* parasites. A negative control (ddH_2_O) and a positive control (sample known to be infected with *Plasmodium* and *Leucocytozoon* parasites) were used in all PCRs to check for possible contamination and efficiency of the PCR. PCR products were run on 2% agarose gel. Positive PCR products were sequenced in both directions with the Big Dye Terminator V3.1 Cycle Sequencing Kit and the ABI PRISM^TM^ 3100 capillary sequencing robot (Applied Biosystems, Foster City, California) or sent for sequencing to Microsynth (Microsynth, Austria). One sample was subjected to cloning of the *18S* ribosomal RNA of parasites following the protocol of a previous study [28].

Chromatograms were checked for possible mixed infections (more than one peak for a base) using the software Geneious Prime 2022.0.2 (https://www.geneious.com, last accessed on 9 November 2022) or Bioedit (https://bioedit.software.informer.com last accessed on 9 November 2022). Sequences were subjected to BLAST searches in MalAvi database [16] and NCBI GenBank database (National Library of Medicine, Bethesda, Maryland, https://www.ncbi.nlm.nih.gov/genbank/, last accessed on 9 November 2022) to determine the lineages. Sequences obtained from infections with no more than two lineages from the same genus were deposited in GenBank OR143042–OR143096 (i.e., sequences from the two bird individuals with mixed infections of possibly four lineages of *H. majoris* were not deposited). 

Genetic pairwise distances between the lineages were calculated in the software MEGA-X: Molecular Evolutionary Genetics Analysis across computing platforms [29].

### 2.5. Deposition of Voucher Preparations

Vouchers of blood preparations (accessions 49493 NS–49495 NS, 49531 NS, 49567 NS, 49568 NS, 49595 NS, 49615 NS–49617 NS) and histological preparations (accessions 49496 NS–49530 NS, 49532 NS–49566 NS, 49569 NS–49594 NS, 49596 NS–49614 NS, 49618 NS–49632 NS, 49642 NS and49643 NS) were deposited to the Nature Research Centre, Vilnius, Lithuania. Vouchers of blood and histological preparations (accessions G466275-G466294) were deposited at the Queensland Museum, Brisbane, Australia.

## 3. Results

### 3.1. Species and Lineage Identification 

**Figure 1:**
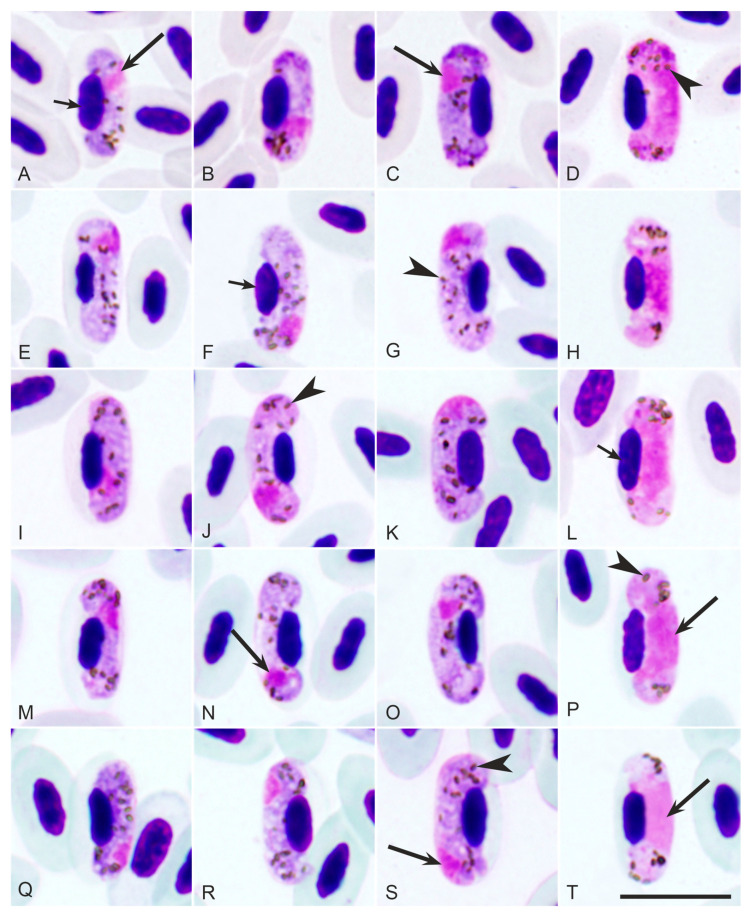
Gametocytes of different *Haemoproteus majoris* lineages found in blood films: hCCF5 (**A**–**D**) in *Fringilla coelebs*, hCWT4 (**E**–**H**) in *Sylvia curruca*, hPARUS1 (**I**–**L**) in *Cyanistes caeruleus*, hPHSIB1 (**M**–**P**) in *Phoenicurus ochruros*, and hWW2 (**Q**–**T**) in *Phylloscopus trochilus*. Note that gametocytes of all lineages are morphologically indistinguishable; the fully grown gametocytes (**C**,**D**,**G**,**H**,**K**,**L**,**O**,**P**,**S**,**T**) of all lineages reach the poles of erythrocytes but do not completely encircle the erythrocyte nuclei, which were displaced laterally. Pigment granules were similar in size, form, and number in gametocytes of all lineages. Long arrow—gametocyte nucleus; short arrow—erythrocyte nucleus; arrowhead—pigment granules. Scale bar: 10 μm.

In total, 42 bird individuals with *H. majoris* infections were dissected (Table 1 and Table S1): 1 *Aegithalos caudatus*, 6 *Fringilla coelebs*, 7 *Cyanistes caeruleus*, 1 *Parus cristatus*, 8 *Parus major*, 1 *Parus montanus*, 2 *Phylloscopus sibilatrix*, 1 *Phylloscopus trochilus*, 1 *Phoenicurus ochruros*, 1 *Phoenicurus phoenicurus*, 3 *Sylvia atricapilla*, 4 *Sylvia communis*, 3 *Sylvia curruca*, and 3 *Sylvia nisoria*. All infections were confirmed by microscopic examination of the blood films with *H. majoris* characteristics (Figure 1), except three individuals (*Aegithalos caudatus*, *Phylloscopus sibilatrix*, and *Phoenicurus phoenicurus*) whose blood films were negative or not usable (from dead birds). One *H. majoris* infection was found in co-infection with *Leucocytozoon* sp. and eleven with other *Haemoproteus* spp., as confirmed by microscopy (Table 1 and Table S1). Parasitemia intensity ranged from 0.01% to 5.1% in single infections of *H. majoris* (Table 1 and Table S1).

The gametocytes present in the hCWT4 infection display the main *H. majoris* characteristics (Figure 1E–H): gametocytes grow around the erythrocyte nuclei but do not encircle them; dumbbell-shaped growing gametocytes are present; and pigment granules are roundish, sometimes oval, of medium to small size, and are randomly scattered. The parasite morphology was the same as described before, and its description is not repeated here [1].

Molecular analysis confirmed the *H. majoris* infections of the dissected birds and revealed five co-infections of different *H. majoris* lineages, two co-infections with other *Haemoproteus* lineages, and eleven co-infections with *Leucocytozoon* lineages (Table 1 and Table S1). In total, seven birds were found infected only with the hCCF5 lineage, eight with hCWT4, twelve with hPARUS1, three with hPHSIB1, and seven with hWW2; these numbers exclude the samples with mixed infection of different *H. majoris* lineages (Table 1 and Table S1). Nine of the identified co-infections by microscopy were not picked up by PCR, and only one lineage was identified in those infections. 

*Haemoproteus* species were identified by microscopic examination in samples with co-infections where exo-erythrocytic stages were found (Table 1).

Birds negative for exo-erythrocytic stages were excluded from this table and are present in Table S1.

*H. majoris* lineages differed from 0.2 to 1.3% (1 to 6 nucleotides) in the 478 bp *cyt b* barcode region (Table 2).

### 3.2. Haemoproteus majoris Exo-Erythrocytic Stages

Only megalomeronts were found during all single *H. majoris* infections. Meronts were only present in samples with mixed infections of *H. majoris* and other *Haemoproteus* species. Megalomeronts were found in 25 of the 42 investigated birds. However, not all organs were collected from 10 of the 17 individuals that were negative for exo-erythrocytic stages (Table 1 and Table S1).

#### 3.2.1. Exo-Erythrocytic Stages Morphology

**Figure 2:**
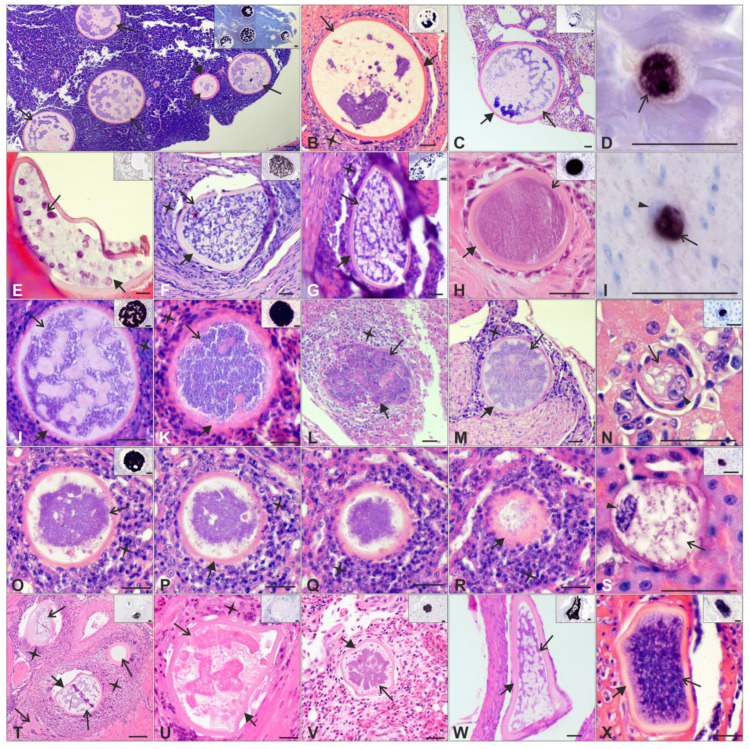
Megalomeronts of different *Haemoproteus majoris* lineages: hCCF5 (**A**–**D**), hCWT4 (**E**–**I**), hPARUS1 (**J**–**N**), hPHSIB1 (**O**–**S**), and hWW2 (**T**–**X**) in haematoxylin and eosin (H&E)-stained sections and their corresponding images after chromogenic in situ hybridization (CISH) treatment (inserts and (**D**,**I**). Megalomeronts were found in pancreas (**A**,**D**), gizzard (**B**,**E**–**I**,**M**,**W**,**X**), lungs (**C**,**K**,**V**), kidneys (**J**,**N**–**S**), liver (**L**), muscles (**T**), and heart (**U**) of their host. Note the variously shaped, interconnected cytomeres in developing megalomeronts (**A**–**C**,**J**,**M**) and the more densely aggregated and connected cytomeres in megalomeronts of hCWT4 and hWW2 (**F**,**G**,**T**,**W**,**X**). Very young megalomeronts (**D**,**I**,**N**,**S**) were found in the infections of four lineages. The host cell nucleus was slightly enlarged and visible in the very young megalomeronts (**D**,**I**,**N**,**S**) but absent in more developed (**A**–**C**,**E**–**H**,**J**,**K**,**M**,**O**–**P**,**T**–**X**) and ruptured (**L**) megalomeronts. Ruptured megalomeronts (**L**) were found in the liver of one individual. One megalomeront was found to appear in serial sections (**O**–**R**) showing how different its morphology and size can be depending on the analyzed section. Megalomeronts were found solitary in the tissues, and sometimes several megalomeronts were found in the same section located close to each other (**A**,**T**). Inflammatory reactions were observed around several megalomeronts (**B**,**F**,**G**,**J**–**M**,**O**–**R**,**T**,**U**). Megalomeronts were surrounded by a thick capsular-like wall, except for the very young ones. Cytomeres were readily visible in stages of advanced development. Long arrow: megalomeront; Short arrow: capsular-like wall; Cross: inflammatory reaction; Arrowhead: enlarged host cell nucleus. Scale bar: 100 μm (**A**,**T**); 25 μm (**B**–**S**,**U**–**X**).

hPARUS1, hCCF5, and hPHSIB1 lineages of *H. majoris* displayed similar morphology to that of megalomeronts (Figure 2A–D,J–S and Figures S1, S3), which were surrounded by a thick capsular-like wall and possessed variously shaped interconnected cytomeres in which merozoites developed. Some megalomeronts in hCWT4 and hWW2 infections were slightly different in their morphology (Figure 2E–I,T–X). Mainly, the cytomeres were present, but they were denser and interconnected more tightly (compare Figure 2F,G,W,X with Figure 2A–C,J). Interestingly, their host species also differed, with hPARUS1 found predominantly in *Parus* spp., hCCF5 in *Fringilla coelebs*, and hCWT4 and hWW2 in *Sylvia* spp. (Table 1 and Table S1).

Some of the megalomeronts found in the gizzard with hCWT4 infection (Figure 2E) developed in the koilin layer and looked degenerated, reacting poorly with the Haemo18*S* probe, while other megalomeronts at a similar stage of growth showed a deep purple CISH signal. Other parasite tissue structures were found in the gizzard with varying shapes, some of them appearing ruptured with very poor to no CISH signal (Figures S2 and S4).

Megalomeront morphology of the same *H. majoris* lineages’ infection did not differ between host species. For example, megalomeronts of hPARUS1 infections found in *Cyanistes caeruleus*, *Parus major*, and *Parus montanus* were of the same morphology (Figure 2J–N and Figure S3).

**Figure 3:**
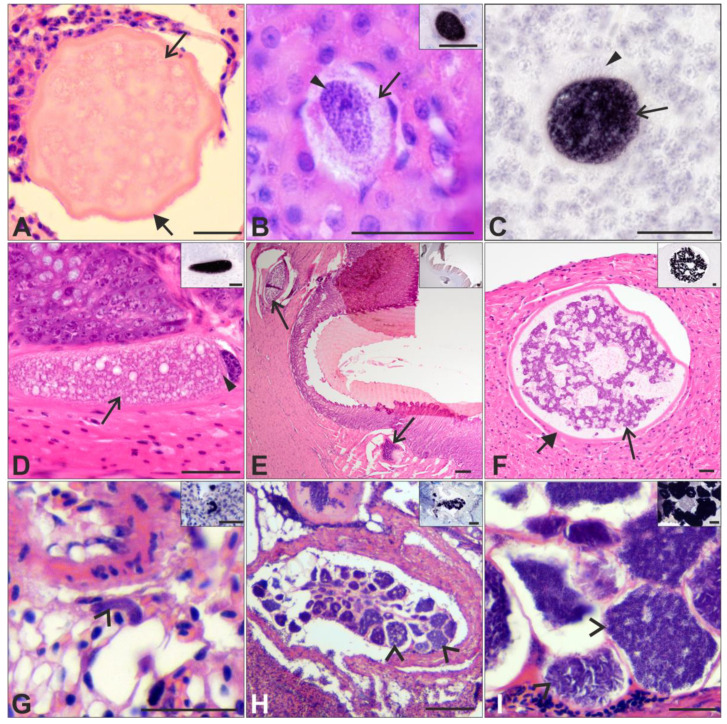
Exo-erythrocytic stages (megalomeronts (**A**–**F**), and meronts (**G**–**I**)) of different *Haemoproteus* species in co-infection with *H. majoris* found in H&E-stained sections and CISH-tested sections (inserts and (**C**)): co-infections of different *H. majoris* lineages—hWW2, hPHSIB1, hPARUS1, and hCWT4—present (**A**) and hWW2 with hPHSIB1 (**B**–**F**) present; *H. majoris* hCCF5 with *H. fringillae* (unknown lineage) (**G**); *H. majoris* hCCF5 with *H. fringillae* and *H. magnus* (unknown lineages) (**H**,**I**). The tissue stages were found in the brain of a *Parus major* (**A**) and a *Phoenicurus ochruros* (**C**); the kidneys of a *Phylloscopus sibilatrix* (**B**); the intestine (**D**), gizzard (**E**), and heart (**F**) of a *Phoenicurus ochruros*; and the lungs of *Fringilla coelebs* (**G**–**I**). The developing megalomeronts (**A**,**E**,**F**) were surrounded by a capsular-like wall. The very young megalomeronts (**B**–**D**) were seen in cells with still the host cell nucleus present, which was not present in developing megalomeronts (**A**,**E**,**F**). Meronts in the lungs were seen either following the capillaries (**G**) or grouped tightly together (**H**,**I**) in a blood vessel of the lungs of *F. coelebs*. Cytomeres were readily visible in megalomeronts (**F**) as well as in developing and maturing meronts (**I**). The CISH signals were deep purple in the developing exo-erythrocytic stages (inserts and (**C**)). Long arrow: megalomeront; short arrow: capsular-like wall; arrowhead: host cell nucleus; opened arrowhead: meront. Scale bar: 25 μm (**A**–**D**,**F**–**I**); 100 μm (**E**).

Very young megalomeronts (less than 20 µm in diameter) were found in 10 birds, 1 in hCCF5, 1 in hCWT4, 5 in hPARUS1, 1 in hPHSIB1 lineage infections, and 2 in *H. majoris* co-infections. The host cell nucleus was present and slightly enlarged (Figure 2D,I,N,S, Figure 3B–D and Figure S3). The host cell nucleus was not seen in more advanced, developing, and mature megalomeronts (Figure 2A–C,E–H,J–M,O–R,T–W and Figures S1–S5).

Inflammatory reactions were observed around some megalomeronts. The inflammatory infiltrates were predominantly lymphohistiocytic, and only occasionally were single heterophils seen. Inflammation was present around seemingly intact megalomeronts, where the intensity could vary between single inflammatory cells (Figure 2B) and several layers of them (Figure 2O–R,T). Adjacent to damaged or ruptured parasitic structures the inflammation was generally severe.

Individuals infected with the same parasite lineage and a similar intensity of parasitemia often featured megalomeronts (Table 1) but not always (Table S1).

#### 3.2.2. Exo-Erythrocytic Stages Site of Development

Considering all the examined lineages of *H. majoris*, the kidneys and the gizzard were most commonly affected by megalomeronts, followed by the lungs and intestine, even though 10 birds had not their intestine and gizzard collected and investigated (Table 1 and Table S1). By considering each lineage of the infection, the lungs, gizzard, and intestine were most often affected in hCCF5 infections, the gizzard in hCWT4 infections, the kidneys and lungs in hPARUS1 infections, and the heart and gizzard in hWW2 infections. In hPHSIB1 infections, only one individual was positive for megalomeronts, with the kidneys affected. In co-infections with several *H. majoris* lineages, the intestine and the brain were most commonly affected (Table 1 and Table S1). 

Very young megalomeronts were found in the brain (Figure 3C, co-infection of hWW2 and hPHSIB1), kidneys (Figure 3B, co-infection of hWW2 and hPHSIB1; Figure 2N and Figure S3I,R,S,Z,AA, hPARUS1 infection), gizzard (Figure 2I and Figure S2N, hCWT4), intestine (Figure S3D and Figure S5H,I, co-infection hWW2 and hPHSIB1), pancreas (Figure 2D, hCCF5), and spleen (Figure S3T, hPARUS1 infection).

The number of *H. majoris* exo-erythrocytic stages present per organ varied per histological section analyzed. In most organs, megalomeronts were only seen sporadically, whereas, in the kidneys, pancreas, and gizzard, megalomeronts were found in greater numbers (up to 8 megalomeronts were seen in a single section) (Figure 2A,T, Figure 3E, Figures S2C and S3K,AD).

#### 3.2.3. Seasonal Occurrence of Exo-Erythrocytic Stages of *H. majoris* hPARUS1

hPARUS1 lineage was sampled in spring, summer, and autumn and displayed exo-erythrocytic stages differently. Megalomeronts were seen in all seasons, however, with different frequencies (Table S2). In spring, the kidneys, lungs, liver, heart, intestine, and gizzard were found affected in 4 to 21 sections out of 13 to 31 sections for the 3 individuals investigated, while in summer megalomeronts were found in 1 to 5 sections out of 9 sections examined for one individual (Table S2). In autumn, mainly the kidneys were found to be affected (with the spleen found to be affected once in 1 bird only), with 21 sections positive for exo-erythrocytic stages out of 304 sections of kidneys investigated from 8 individuals (Table S2). Ten times more slides were thus investigated to find approximately the same number of positive sections for the kidneys between individuals collected in spring and autumn (Table S2). The other organs were not found affected by megalomeronts in autumn, even after investigating more than 100 sections, while in spring and summer, around 10 investigated sections reported a high number of exo-erythrocytic stages (Table S2).

In hPARUS1 single infections of *Haemoproteus*, very young megalomeronts were found in two individuals in spring and in four individuals in autumn. More of these very young megalomeronts were found in individuals in autumn (26 found in 21 positive sections out of 304 sections of the kidneys investigated) than in spring (3 found in 20 positive sections out of 31 sections of the kidneys investigated). In spring, more than 1 big megalomeront was found per section, adding up to more than 90 megalomeronts (without distinguishing if it is a different megalomeront from the previous section) in the kidneys in 20 positive sections out of 31, while in autumn, 11 big megalomeronts were found in the 304 investigated sections of kidneys.

#### 3.2.4. Exo-Erythrocytic Stages in *H. majoris* Co-Infections with Several of Its Lineages

Five samples were found to be co-infected with several lineages of *H. majoris*, as determined by PCR-based testing and the obtained *cyt b* sequences (Table 1 and Table S1). The megalomeront found in the brain of one individual co-infected with several *H. majoris* lineages (Figure 3A) could not be confirmed as *Haemoproteus* as the structure was absent in the CISH-tested section. Only eosinophilic staining and no basophilic staining was observed, which could indicate the occurrence of a degenerating megalomeront.

Very young megalomeronts were found in the brain, kidneys, and intestine of two individuals, each co-infected with hWW2 and hPHSIB1. The very young megalomeront found in the intestine was bigger (~96 µm in largest diameter) than the other very young megalomeronts (<20 µm in largest diameter). However, the slightly enlarged host cell nucleus was still visible in the former (Figure 3D).

Some of the megalomeronts found in sample ‘20 Sp’ co-infected with the lineages hPHSIB1 and hWW2 (Figure 3E) looked similar to those found in single infections with hCWT4 and hWW2 (Figure 2F,G,W,X).

### 3.3. Exo-Erythrocytic Stages of Other Haemoproteus species

Small meronts (up to 25 µm) were found in a *Fringilla coelebs* (sample ‘1 Sp’) where microscopic examination identified the presence of *H. fringillae* in blood films. Positive CISH signals with the Haemo18*S* probe were observed for these meronts, confirming their *Haemoproteus* origin (Figure 3G insert). However, as no other sample investigated with single infection of *H. majoris* revealed small meronts, these stages likely belong to another *Haemoproteus* species, most likely *H. fringillae*, and not *H. majoris*.

Big meronts (up to 60 µm) were found in *F. coelebs* (‘3 Sp’) in a blood vessel of the lungs, and positive CISH signals were observed for the corresponding parasites seen in H&E-stained sections (Figure 3H,I). The meronts were tightly grouped in a blood vessel, forming a cluster well visible under the microscope. Individual meronts of this cluster were at different stages of development, with merozoites well visible in some meronts, which showed little CISH signal, whereas other developing meronts had deep purple CISH signals (Figure 3I insert). This indicates the asynchronous maturation of meronts and the asynchronous release of merozoites. Microscopic examination identified the presence of three other *Haemoproteus* species in the blood films of the *F. coelebs* individual ‘3 Sp’ (*H. fringillae*, *H. magnus,* and *H.* sp.—Table 1 and Table S1). It was not possible to determine the species identity of the *Haemoproteus* meronts found. However, as none of the *H. majoris* single-infected birds were found with such meronts, the latter are most likely not *H. majoris* but belong to one of the other *Haemoproteus* species present in the co-infection. 

## 4. Discussion

The main result of this study is the report and comparative analysis of the exo-erythrocytic stages (megalomeronts) for five lineages (hCCF5, hCWT4, hPARUS1, hPHSIB1, and hWW2) of *H. majoris* in different avian host species. Megalomeronts developed in the examined bird individuals during single infections. This is the first comparative study that addresses the exo-erythrocytic development of different lineages of one *Haemoproteus* species in different avian hosts, providing opportunities to address possible patterns of development in avian haemoproteids. This study also identified the lineage hCWT4 as another lineage of *H. majoris*; this contributes to better understanding the intra-species genetic diversity of this widespread *Haemoproteus* species. 

The lineages hPARUS1, hPHSIB1, hCCF5, and hWW2 were identified as *H. majoris* by Križanauskienė et al. [19], but the parasitemia intensity was too low in the sample infected with hCWT4 to describe and identify the species [19]. In our study, the microscopic examination of blood films of all birds revealed *H. majoris* infections, including all hCWT4 infections and the co-infections with several other *Haemoproteus* species. 

Twelve birds were infected with *H. majoris* lineage hPARUS1, and megalomeronts were found to develop in the kidneys of eight, in the lungs of three, in the liver of two, and in the heart, spleen, pancreas, and gizzard of one bird (Table 1 and Table S1). The megalomeronts were found in different bird species (*P. major*, *C. caeruleus*, and *P. montanus*) and their structure was similar to that of the megalomeronts previously reported in the kidneys, liver, lungs, and spleen of a single *P. major* [9]. This confirms the kidneys as the main site of development of *H. majoris* hPARUS1.

A previous report identified the kidneys and the intestine as sites of development of *H. majoris* lineage hPHSIB1 [12]. In our study, the lineage hPHSIB1 was found in three bird species, with megalomeronts observed only in the kidneys of one *P. sibilatrix* (Table 1 and Table S1). It should be noted that the intestine and the gizzard were not collected for histological purposes for two individuals (*S. atricapilla* and *P. sibilatrix*), while the third individual (*Aegithalos caudatus*) was found dead and no information on gametocytes was available. It is thus difficult to know if the intestine is one of the organs most commonly affected by hPHSIB1 infections or if this is only the case for the kidneys. No individuals were found infected with the lineage hPHYBOR04 of *H. majoris*, for which megalomeronts were described in the kidneys of a *Turdus pilaris* [9]. As was the case with lineage hPARUS1, only megalomeronts were found in all examined birds infected with the lineages hPHSIB1 (this study, [12]) and hPHYBOR04 [9].

*H. majoris* lineage hCCF5 was mostly found in *Fringilla coelebs*. Megalomeronts were found to develop mainly in the lungs, pancreas, and gizzard (Table 1 and Table S1). Only one out of seven individuals showed megalomeronts in the kidneys. In this regard, hCCF5 seems different from the lineages hPARUS1, hPHSIB1, or hPHYBOR04, whose tissue stages mainly developed in the kidneys (this study, [9]). The morphology of the hCCF5 exo-erythrocytic stages was highly similar to the megalomeronts of the lineages hPARUS1, hPHYBOR04, and hPHSIB1 found in this and previous studies [9,12]. In all these megalomeronts, a capsular-like wall surrounded the parasite, in which interconnected cytomeres were seen to develop (Figure 2A–C). 

*H. majoris* parasites of the lineages hCWT4 and hWW2 were mostly found affecting the gizzard (found in 5 individuals), even though the gizzard and intestine were not collected in 9 out of 15 birds. The kidneys were sampled and examined from all birds, but no megalomeronts were found during hCWT4 infections (eight individuals) and were seen only in one out of seven hWW2 infections. This differs compared with the previously reported sites of development of megalomeronts in hPARUS1, hPHSIB1, and hPHYBOR04 infections (this study, [9,12]). The morphology of some megalomeronts found in hCWT4 and hWW2 infections was slightly different from megalomeronts of other *H majoris* lineages. Mainly, the cytomeres were interconnected more tightly (similar to a spiderweb), while megalomeronts of hPARUS1, hPHSIB1, hCCF5, and hPHYBOR04 infections possessed interconnected cytomeres of a bigger size (compare Figure 2F,G,W,X with Figure 2A–C, Figures S2,S4 vs. S1,S3). This could reflect a lineage variation or a difference in the stage of development of the megalomeronts. The megalomeronts found in hCWT4 and hWW2 infections were more irregularly shaped compared with the typical roundish form of megalomeronts (compare Figure 2E–G,T–X with Figure 2A–C,J–M,O–R and Figures S2,S4 vs. S1,S3). This difference in shape could be due to the different locations in the organs. hCWT4 megalomeronts were found in the muscular layers of the gizzard, where they are exposed to varying pressures during peristaltic contractions of the organ. This could explain the irregular shapes of the megalomeronts observed. Megalomeronts in hCWT4 infections were also found to develop in the koilin cuticle of the gizzard, a thick protective layer on the inside of the gizzard that is continuously worn out and where no nutrients are delivered. This could be detrimental to the development of megalomeronts, as their growth might be altered. It is to be noted that none of the megalomeronts found in the koilin cuticle were mature (Figure 2E), and the CISH signals were only light purple, suggesting that the development of the parasite had stopped (low expression of RNA while the stage was not mature either).

Megalomeronts with fully developed merozoites showed barely any CISH signal (Figure S3P,AO), compared with the young and developing stages (Figure 2 and Figure 3 vs. Figures S1–S5), a phenomenon already observed in other *Haemoproteus* parasites and suggesting low ribosomal content in mature stages [30]. The very young megalomeronts were found more easily due to their deep purple CISH signals, facilitating their search in the corresponding H&E-stained sections. Their small sizes and location in host cells with the host cell nuclei present but slightly enlarged were striking compared with previous reports of *Haemoproteus* parasites [7,8,9,10,11,12,13,15]. These very young megalomeronts were found in different host species infected with different *H. majoris* lineages (Figure 2D,I,N,S; Figures S3I,R–T,W–AA, Figure S5H,I). In the infections with hPARUS1, very young megalomeronts were found in the kidneys of several individuals collected in different seasons. They were more often observed in autumn than in spring in positive sections, while developing and mature megalomeronts were more often found in spring.

Megalomeronts of *H. majoris* hPARUS1 were found in three bird species, two of which were found at different times of the year. *Parus major* were found infected with hPARUS1 twice in spring (one negative for megalomeronts in this study and one positive in [9]), once in summer (positive in this study), and twice in autumn (positive in this study), while *Cyanistes caeruleus* was sampled twice in the spring (positive in this study) and five times in autumn (two positive and three negative in this study). The individuals sampled in spring contained the largest number of megalomeronts, with up to four organs affected, while fewer individuals were affected by megalomeronts in autumn, with only one to two organs affected (and at a lesser intensity). These seasonal differences in the maturation of the tissue stages might result from a different parasite-load strategy in the host. In Europe, spring is characterized by the active transmission of *Haemoproteus* parasites [1], and the increase in parasitemia in blood, which is readily visible in blood films. Mature megalomeronts are expected to be more numerous in spring, as merozoites are actively produced and released into circulation. In comparison, parasitemia usually markedly decreases in autumn and is barely present during winter. This seasonality in parasitemia is directly related to exo-erythrocytic development in haemoproteids and should result in less numerous megalomeronts in organs, lessening the burden on the host until the relapse of infection in the following spring [1]. It is still unknown under which stage (unicellular hypnozoite-like parasites or dormant megalomeronts at early development or advanced stage) *Haemoproteus* parasites are present in their host during winter, as gametocytes are rarely seen in blood films. A small number of young megalomeronts might be enough to initiate exo-erythrocytic development and relapse in spring, but their size and presence might also harm the host as inflammatory reactions are observed. Very young megalomeronts, due to their smaller size, might have a lesser influence on the host, but as their CISH signals were deep purple, active development and growth still occur. It is unknown whether the parasite can slow down its development and persist during winter.

*Haemoproteus majoris* is a generalist parasite [21] with six lineages differing by 1 to 6 bp (0.2–1.3 %) in the *cyt b* barcoding sequence. The lineage hCWT4 is closer to hWW2 and hPHYBOR04, with a 1 bp difference (0.2 %), whereas hPARUS1, hPHSIB1, and CCF5 have more differences from the other lineages (differences range from 2 to 6 bp). It seems probable that megalomeronts are the main and probably only tissue stage during *H. majoris* infections. The morphological similarities of megalomeronts and the sites of their development seem to reflect their genetic distances (hCWT4/hWW2 vs. hCCF5/hPARUS1). 

This is the first study which analyzed more than two lineages belonging to one *Haemoproteus* species in regard to their exo-erythrocytic development. It is thus difficult to speculate if the slight differences in morphology and site of development between the *H. majoris* lineages are due to the generalist features of this parasite or are due to lineage-specific features, or both. It would be interesting to study additional multi-lineage species, such as *H. tartakovskyi, H. lanii*, and *H. balmorali*, to explore intraspecific variation of exo-erythrocytic development. Only one parasite closely related to *H. balmorali*, *H. attenuatus,* has been reported to develop meronts in the lungs of its host [14]. An investigation into the exo-erythrocytic stages of other closely related species, which are genetically closely related, would help to better understand if the genetic distances can predict patterns of the exo-erythrocytic development or if the development is only species-specific.

Differences in parasitemia intensity were small in most sampled birds, with parasitemia intensity mostly around 1% (Table 1). Among individuals infected with the same lineage and showing the same intensity of parasitemia, some contained megalomeronts but others did not (Table S1). Similar results were previously reported during *H. pastoris* infection in *Sturnus vulgaris* [13]. Mainly, no trend for megalomeront development was determined among the individuals with parasitemia ranging from 1 to 26%, with the exception of one individual with 10% parasitemia, for which no megalomeronts were found [13]. Parasitemia intensity was not associated either with the number of affected organs by megalomeronts. In *H. pastoris*, nine organs were reportedly affected by the parasites [13], while for all examined *H. majoris* lineages and hosts, megalomeronts were found in ten organs (this study, [9,12]). 

Finally, two birds with *H. majoris* infections were found to be co-infected with other *Haemoproteus* spp., and other exo-erythrocytic stages were found. These exo-erythrocytic stages were meronts of small sizes following the capillaries of the lungs (Figure 3G), and they were similar to meronts found in *H. dumbbellus* [30] of one individual or bigger and clustered together in a blood vessel of the lungs (Figure 3H,I) of the second individual. No meronts were previously reported in *H. majoris* infections, only megalomeronts (this study, [9,12]). Deep purple in situ CISH signals were observed in the meronts in the two individuals, confirming their *Haemoproteus* identity (Figure 3G–I inserts), and the microscopic examination of the blood smears identified co-infections in both cases with *H. fringillae*. It should be noted that the second individual was also infected with other *Haemoproteus* spp., aside from *H. majoris* and *H. fringillae*. It is most likely that these meronts are not of *H. majoris* but might very well be of *H. fringillae*. This observation shows the difficulties in the research on the exo-erythrocytic development of haemosporidians during co-infections, which predominate in wildlife and calls for the development of more specific in situ diagnostic probes (e.g., on a species or even lineage level), which would increase the resolution of exo-erythrocytic stages belonging to different parasite species.

## 5. Conclusions

This study extends knowledge about the lineage diversity of the widespread *H. majoris* haemosporidian parasite by adding the lineage hCWT4 to this morphospecies. Only megalomeronts were observed developing in all examined single *H. majoris* infections, strongly suggesting that meronts might be absent in this parasite. Megalomeronts were also found in mixed infections of several *H. majoris* lineages, while meronts were only found in cases of mixed infections of *H. majoris* and other *Haemoproteus* species. The following findings are worth more attention.

The organs were found to be differently affected by *H. majoris* megalomeronts, with hPARUS1 megalomeronts most commonly found in the kidneys; hCCF5 in the lungs, intestine, and kidneys; hCWT4 in the gizzard; and hWW2 in the heart and gizzard. Megalomeronts were also found in those organs for the other lineages (but at a lower intensity). The morphology of all reported *H. majoris* megalomeronts was mostly similar in all lineages, with some minor differences in hCWT4 and hWW2 infections. Very young megalomeronts were found for four of the *H. majoris* lineages. The parasitemia intensity could not be associated with the presence of megalomeronts in organs. However, megalomeronts were more often found in birds from spring than from autumn, with more developing and mature megalomeronts in spring and more very young stages in autumn. It would be interesting to investigate other generalist and specialist parasite species in different avian hosts across different seasons to better understand the pattern of their development and to make sure how the exo-erythrocytic development depends on the parasite lineage, the host species, and the season. It would also be interesting to see if very young megalomeronts can be found in the host during the winter period, possibly as dormant stages responsible for relapses in spring.

## Figures and Tables

**Table 1:** Data on individuals infected with *Haemoproteus majoris* showing exo-erythrocytic stages. Information is organized by lineage of infection and data are provided for the season of collection, parasitemia, host species, parasite identification (through microscopy and PCR), and organs in which the exo-erythrocytic stages were found both in haematoxylin and eosin (H&E)-stained sections and the sections treated with chromogenic in situ hybridization (CISH) with the probe Haemo18*S*.

Identity No. ^#^	Parasite -Mia (%)	Host Species	Parasite Species and Cytochrome *b* Lineages	Tissue Stages Seen in H&E and CISH Sections ^‡^								
				He	Lu	Li	Sp	Ki	Br	Mu	Int	Gi
1 Sp	0.7 ^a^	*Fringilla coelebs*	*H. majoris* (hCCF5), *Haemoproteus fringillae* ^b^, *Leucocytozoon* sp. (lBRAM3) ^c^	+	H	-	-	-	-	-	na	na
3 Sp	2.8 ^a^	*F. coelebs*	*H. majoris* (hCCF5), *H. fringillae* ^b^, *Haemoproteus magnus* ^b^, *Haemoproteus* sp. ^b^	-	+/H	-	-	-	-	-	na	na
19 Sp	0.2	*F. coelebs*	*H. majoris* (hCCF5)	-	-	-	-	-	-	-	-	+ ^e^
27 Sp	0.2 ^a^	*F. coelebs*	*H. majoris* (hCCF5), *Mixed infection* ^b^	-	+	-	-	+	-	-	+	+
28 Sp	0.1 ^a^	*F. coelebs*	*H. majoris* (hCCF5), *L.* sp. (lEMCIR02) ^c^, *Mixed infection* ^b^	-	-	-	-	-	-	-	+ ^f^	+
3 Su	0.02	*Parus cristatus*	*H. majoris* (hCCF5)	-	-	-	-	-	-	-	+ ^e,f^	-
6 Sp	1.3	*Sylvia communis*	*H. majoris* (hCWT4)	-	-	-	-	-	-	+ ^d^	na	na
18 Sp	1.6	*Sylvia curruca*	*H. majoris* (hCWT4)	-	-	-	-	-	-	-	+	+
21 Sp	1.1	*S. curruca*	*H. majoris* (hCWT4)	-	-	-	-	-	-	-	-	+ ^e^
22 Sp	1	*S. curruca*	*H. majoris* (hCWT4)	-	-	-	-	-	-	-	-	+
7 Sp	1.2	*Cyanistes caeruleus*	*H. majoris* (hPARUS1)	+	+	+	-	+	-	-	na	na
23 Sp	5.1	*C. caeruleus*	*H. majoris* (hPARUS1), *L.* sp. (lPARUS14) ^c^	-	+	+	-	+	-	-	-	+
2 Au	0.1	*C. caeruleus*	*H. majoris* (hPARUS1)	-	-	-	-	+	-	-	-	-
4 Au	0.1	*C. caeruleus*	*H. majoris* (hPARUS1), *L.* sp. (PARUS4) ^c^	-	-	-	-	+ ^e^	-	-	-	-
2 Su	0.5	*Parus major*	*H. majoris* (hPARUS1)	-	+	-	-	+	-	-	+ ^e, f^	-
6 Au	0.07	*P. major*	*H. majoris* (hPARUS1)	-	-	-	+ ^e^	+	-	-	-	-
7 Au	0.01	*P. major*	*H. majoris* (hPARUS1), *L.* sp. (lPARUS18) ^c^	-	-	-	-	+ ^e^	-	-	-	-
8 Au	0.03	*Parus montanus*	*H. majoris* (hPARUS1)	-	-	-	-	+	-	-	-	-
9 Sp	NEG	*Phylloscopus sibilatrix*	*H. majoris* (hPHSIB1)	-	-	-	-	+	-	-	na	na
4 Sp	0.6	*P. major*	*Haemoproteus majoris* (hWW2/hCWT4/hPARUS1/hPHSIB1)	-	-	+	-	-	-	-	na	na
1 Su	0.1	*P. major*	*H. majoris* (hWW2/hCWT4/hPARUS1/ hPHSIB1)	-	-	-	-	-	+ ^d^	-	-	-
12 Sp	6.3 ^a^	*Sylvia atricapilla*	*H. majoris* (hWW2), *Haemoproteus parabelopolskyi* ^b^, *L.* sp. (lSYAT22) ^c^, *Leucocytozoon majoris* ^b^	+	+	-	-	-	-	+	na	na
24 Sp	4.7 ^a^	*S. atricapilla*	*H. majoris* (hWW2), *H.* sp.1 ^b^, *H. parabelopolskyi* ^b^, *H.* sp 2. ^b^, *L.* sp. (lCOCOE09) ^c^	+	-	-	-	+	-	-	-	+
17 Sp	0.7	*Phylloscopus sibilatrix*	*H. majoris* (hWW2 and hPHSIB1)	-	-	-	na	+	-	-	+ ^e^	-
29 Sp	0.6	*Phylloscopus trochilus*	*H. majoris* (hWW2)	-	-	-	-	-	-	-	-	+
20 Sp	1.1	*Phoenicurus ochruros*	*H. majoris* (hPHSIB1 and hWW2 ^g^)	+	-	-	-	-	+	-	+	+

^#^ “Sp“ spring; “Su“ summer; “Au“ autumn. ^a^ Overall parasitemia intensity for all parasites present (no distinction between the species present in co-infections). ^b^ Parasite only seen during microscopy but not detected by PCR; ^c^ parasite not observed during microscopic examination of blood films, so only PCR results are available. ^‡^ The trachea, esophagus, testes, or ovaries were negative for all individuals and excluded from this table; He: heart; Lu: lungs; Li: liver; Sp: spleen; Ki: kidneys; Br: brain; Mu: skeletal muscle; Int: intestine; Gi: gizzard; “-“ samples negative for *Haemoproteus* exo-erythrocytic stages in both H&E and CISH sections; “+” samples positive for *H. majoris* exo-erythrocytic stages in both H&E and CISH sections; “na” organ not collected, or the sections are not present for the analysis of tissue stages; ”H” samples positive for exo-erythrocytic stages of other *Haemoproteus* spp. in both H&E and CISH sections; ^d^ parasite was not present in the consecutive histological section, so not tested by CISH; ^e^ stage present only in CISH; ^f^ stages found in the pancreas, not the intestine. ^g^ Lineage obtained by cloning [28].

**Table 2:** Pairwise distances in percentage between the different *Haemoproteus majoris* lineages in the 478 bp cytochrome *b* sequence.

Lineage	hCWT4	hPARUS1	hPHSIB1	hPHYBOR04	hWW2
hCCF5	0.6	0.6	1.3	0.8	0.8
hCWT4		0.4	0.6	0.2	0.2
hPARUS1			1.0	0.6	0.6
hPHSIB1				0.8	0.4
hPHSYBOR04					0.4

## Data Availability

The generated sequence data were deposited in the NCBI GenBank database. The parasite voucher preparations (blood and histological preparations) are available at Nature Research Centre, Vilnius, Lithuania, and at the Queensland Museum, Brisbane, Australia, upon request.

## Supplementary Materials

The following supporting information can be downloaded at https://www.mdpi.com/article/10.3390/pathogens12070898/s1. Figure S1: Megalomeronts of *Haemoproteus majoris* lineage hCCF5 in *Fringilla coelebs*; Figure S2: Megalomeronts of *Haemoproteus majoris* lineage hCWT4 in *Sylvia communis* and *Sylvia curruca*; Figure S3: Megalomeronts of *Haemoproteus majoris* lineage hPARUS1 in *Cyanistes caeruleus*, *Parus major*, and *Parus montanus*; Figure S4: Megalomeronts of *Haemoproteus majoris* lineage hWW2 in *Sylvia atricapilla*; Figure S5: Megalomeronts of *Haemoproteus majoris* found in co-infections with several of its lineages; Table S1: Data on all individuals with *Haemoproteus majoris* infections investigated for exo-erythrocytic stages; Table S2: Data on all individuals with *Haemoproteus majoris* hPARUS1 infections investigated for exo-erythrocytic stages numbers.
