# Supplementary material for: Comparative Analysis of the Exo-Erythrocytic Development of Five Lineages of Haemoproteus majoris, a Common Haemosporidian Parasite of European Passeriform Birds
Source: Pathogens. 2023 Jun 30;12(7):898. doi: 10.3390/pathogens12070898 (PMC10386383; doi:10.3390/pathogens12070898)
Supplement: Supplementary file 1 [file pathogens-12-00898-s001.zip › Figure S1.pdf]

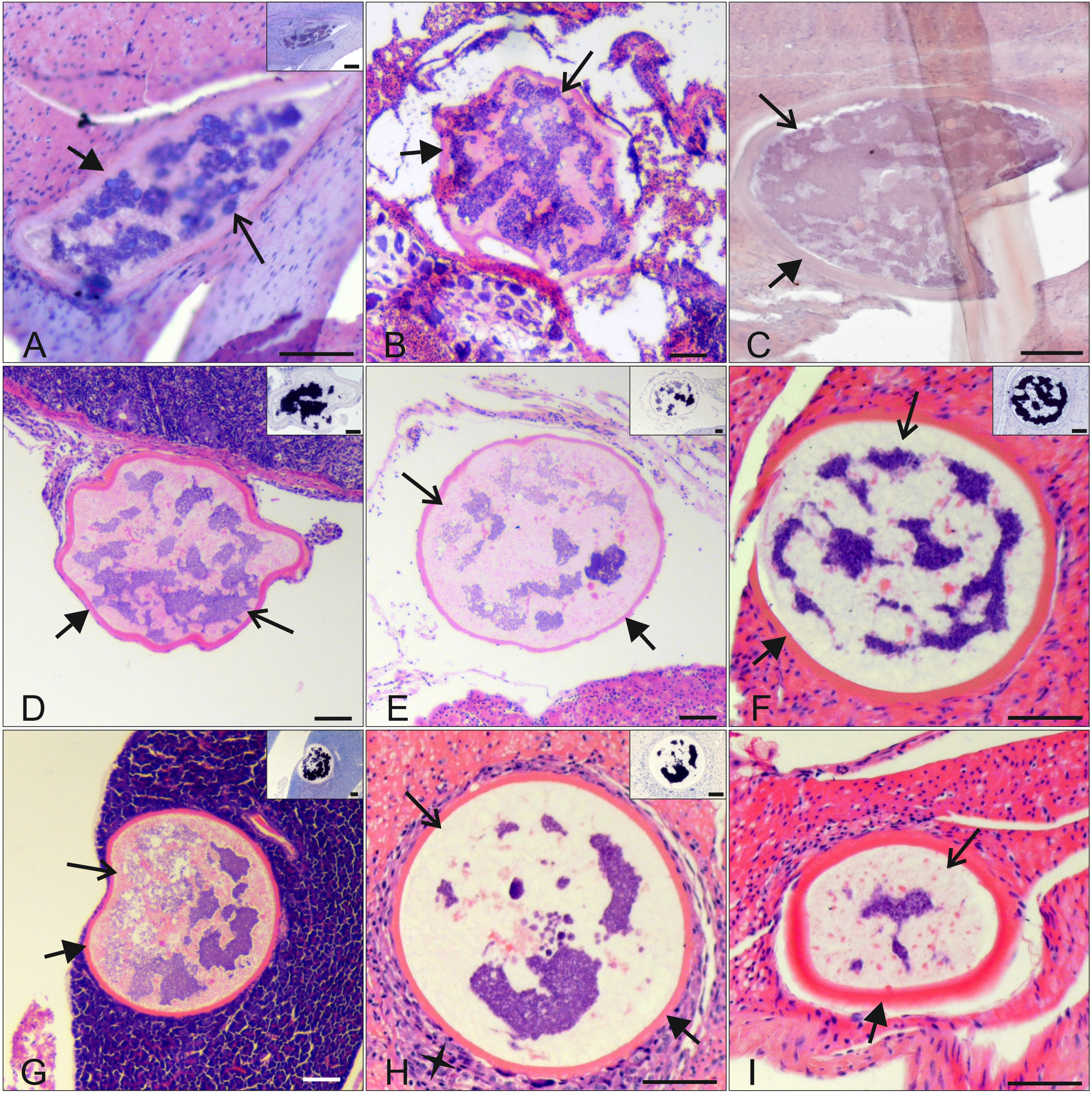

Figure S1. Megalomeronts of *Haemoproteus majoris* lineage hCCF5 in *Fringilla coelebs* in hematoxylin and eosin (H&E) stained-sections and their corresponding images after chromogenic in situ hybridization (CISH) treatment (inserts and C): in '1 Sp' heart (A), '3 Sp' lungs (B), '19 Sp' gizzard (C), '27 Sp' intestine/ pancreas (D), kidneys (renal capsule/ perirenal at) (E), gizzard (F), and '28 Sp' pancreas (G) and gizzard (H, I). Note the variously shaped interconnected cytomeres in developing megalomeronts (A-I). Megalomeronts were surrounded by a thick capsular-like wall. Cytomeres were readily visible. Long arrow: megalomeront; short arrow: capsular-like wall; cross: inflammatory reaction. Scale bar: 50 µm.
