# Supplementary material for: Comparative Analysis of the Exo-Erythrocytic Development of Five Lineages of Haemoproteus majoris, a Common Haemosporidian Parasite of European Passeriform Birds
Source: Pathogens. 2023 Jun 30;12(7):898. doi: 10.3390/pathogens12070898 (PMC10386383; doi:10.3390/pathogens12070898)
Supplement: Supplementary file 1 [file pathogens-12-00898-s001.zip › Figure S2.pdf]

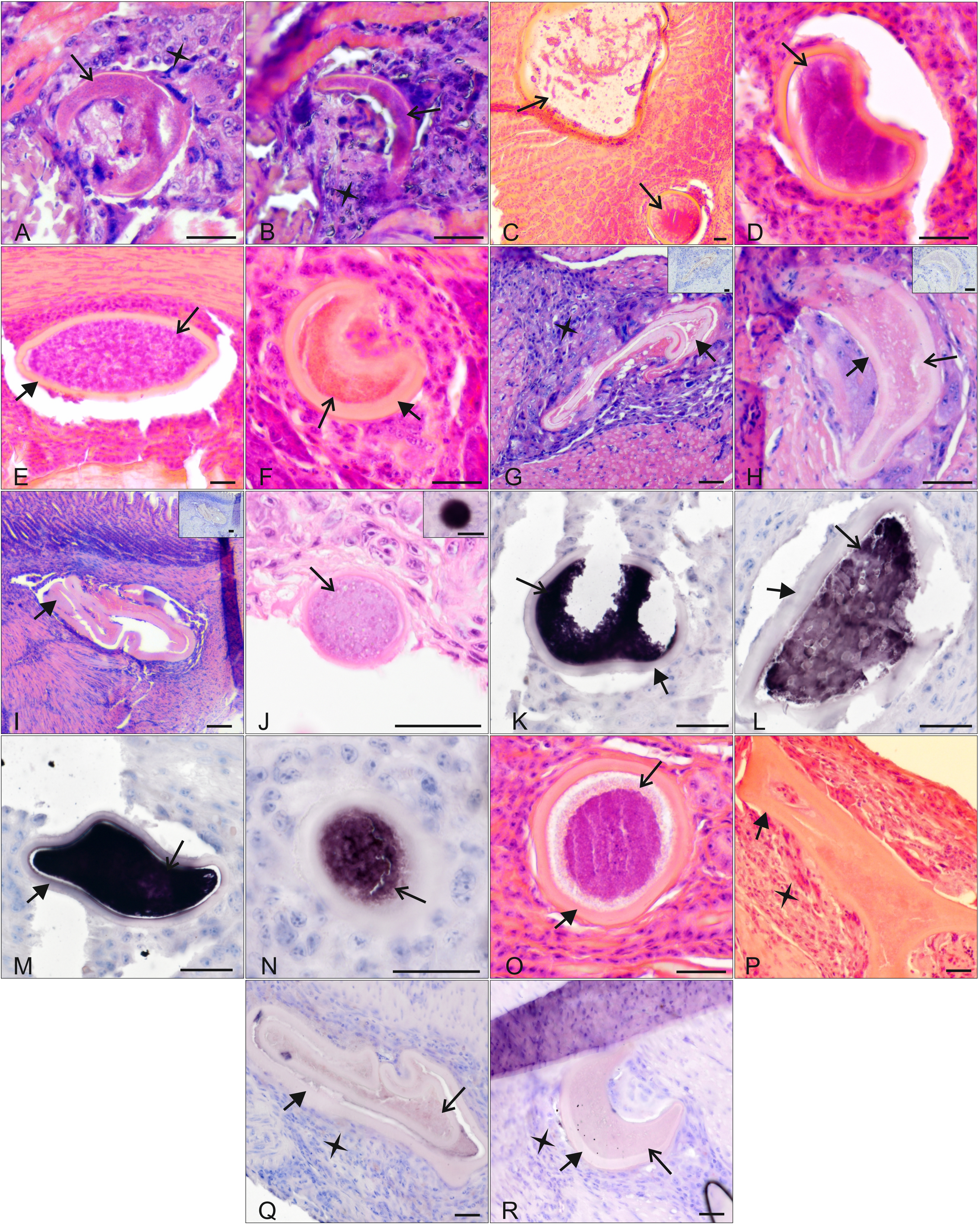

Figure S2. Megalomeronts of *Haemoproteus majoris* lineage hCWT4 in *Sylvia communis* (A, B) and *Sylvia curruca* (C–R) in hematoxylin and eosin (H&E) stained-sections and their corresponding images after chromogenic *in situ* hybridization (CISH) treatment (inserts and J–M, P, Q): in '6 Sp' muscle (A, B), '18 Sp' gizzard (C–H, J–N), intestine (I), '21 Sp' gizzard (O–R). Note the more densely located and connected cytomeres in developing megalomeronts (B, D, K). Young megalomeronts were observed with deep purple CISH signals (I, J, L) and one very young megalomeront (M) was found only in the CISH section. Ruptured megalomeronts (F, H, P) were found in gizzards. Megalomeronts were found alone in the tissues and sometimes several were found very close to each other in the same section (B). Inflammatory reactions were observed around several megalomeronts (A, B, F, O–Q). Megalomeronts were surrounded by a thick capsular-like wall. Cytomeres were readily visible. Long arrow: megalomeront; short arrow: capsular-like wall; cross: inflammatory reaction. Scale bar: 25 μm.
