# Supplementary material for: Comparative Analysis of the Exo-Erythrocytic Development of Five Lineages of Haemoproteus majoris, a Common Haemosporidian Parasite of European Passeriform Birds
Source: Pathogens. 2023 Jun 30;12(7):898. doi: 10.3390/pathogens12070898 (PMC10386383; doi:10.3390/pathogens12070898)
Supplement: Supplementary file 1 [file pathogens-12-00898-s001.zip › Figure S3.pdf]

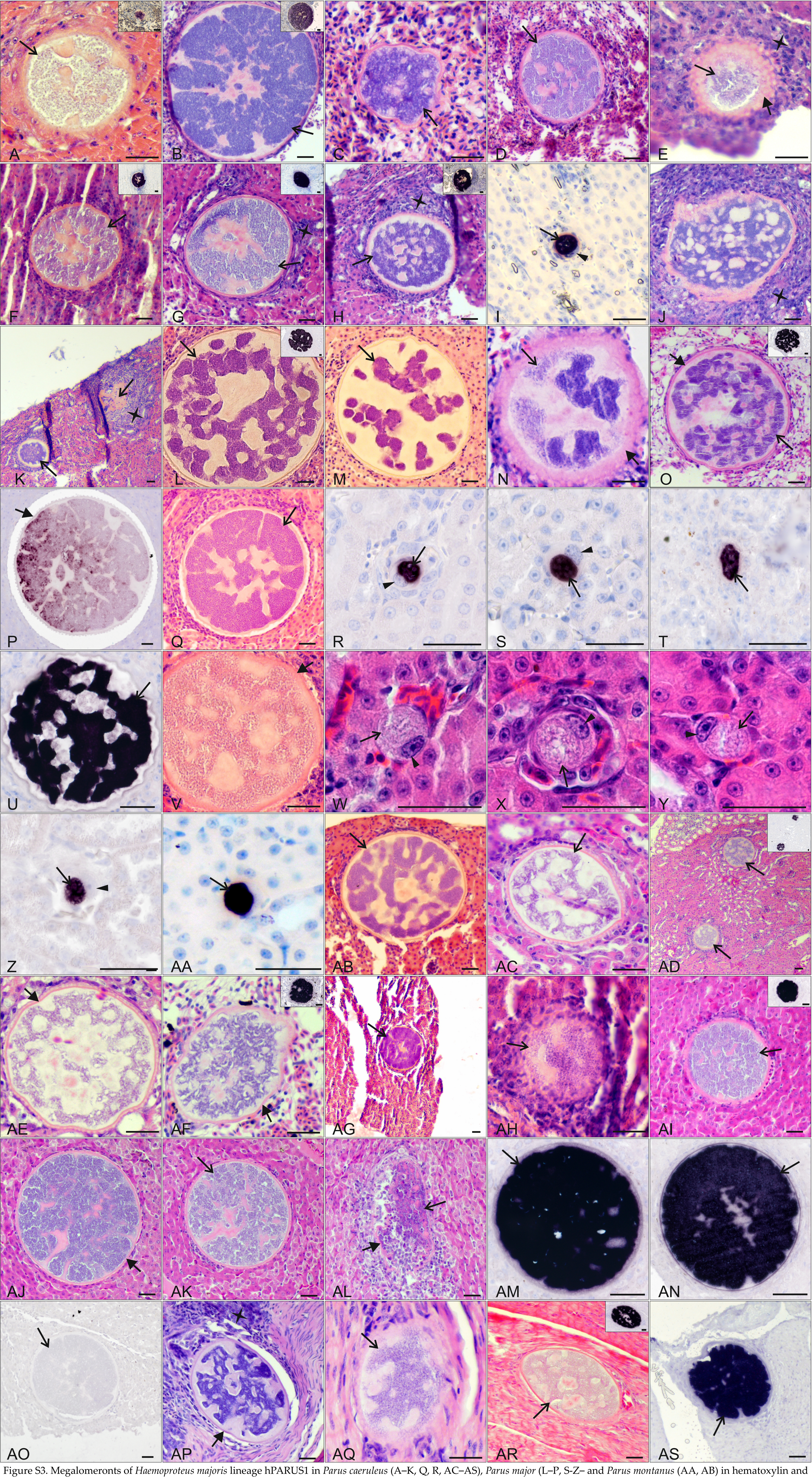

Figure S3. Megalomeronts of *Haemoproteus majoris* lineage hPARUS1 in *Parus caeruleus* (A–K, Q, R, AC–AS), *Parus major* (L–P, S-Z– and *Parus montanus* (AA, AB) in hematoxylin and eosin (H&E) stained-sections and their corresponding images after chromogenic *in situ* hybridization (CISH) treatment (inserts and I, P, R–U, Z, AA, AM–AO, AS): in '7 Sp' heart (A), lungs (C–D), liver (E, F), kidneys (G–K), '2 Su' kidneys (L–M), lungs (N–O), pancreas (P), '2 Au' kidneys (Q), '4 Au' kidneys (R), '6 Au' kidneys (S), spleen (T), kidneys (U–Y), '7 Au' kidneys (Z), '8 Au' kidneys (AA, AB), '23 Sp' kidneys (AC–AE), lungs (AF), liver (AG–AO), gizzard (AP–AS). Note the variously shaped interconnected cytomeres in developing megalomeronts (A–H, L–Q, U, V, AB–Ak, AM–AS). Very young megalomeronts (I, R–T, W–AA) were observed in H&E and CISH sections. A ruptured megalomeront (AL) was found in liver. The host cell nucleus was slightly enlarged and visible in the very young megalomeronts (I, R, S, W–Z) but absent in the more developed (A–H, J-Q, U, V, AB–Ak, AM–AS) and ruptured (AL) megalomeronts. Megalomeronts were found alone in the tissues and sometimes several were found very closely to each other in the same section (K, AD). Inflammatory reactions were observed around several megalomeronts (E, H, J, K, AP). Megalomeronts were surrounded by a thick capsular-like wall. Cytomeres were readily visible. Long arrow: megalomeront; short arrow: capsular-like wall; cross: inflammatory reaction; arrowhead: enlarged host cell nucleus. Scale bar: 25 µm.
