# Supplementary material for: Comparative Analysis of the Exo-Erythrocytic Development of Five Lineages of Haemoproteus majoris, a Common Haemosporidian Parasite of European Passeriform Birds
Source: Pathogens. 2023 Jun 30;12(7):898. doi: 10.3390/pathogens12070898 (PMC10386383; doi:10.3390/pathogens12070898)
Supplement: Supplementary file 1 [file pathogens-12-00898-s001.zip › Figure S4.pdf]

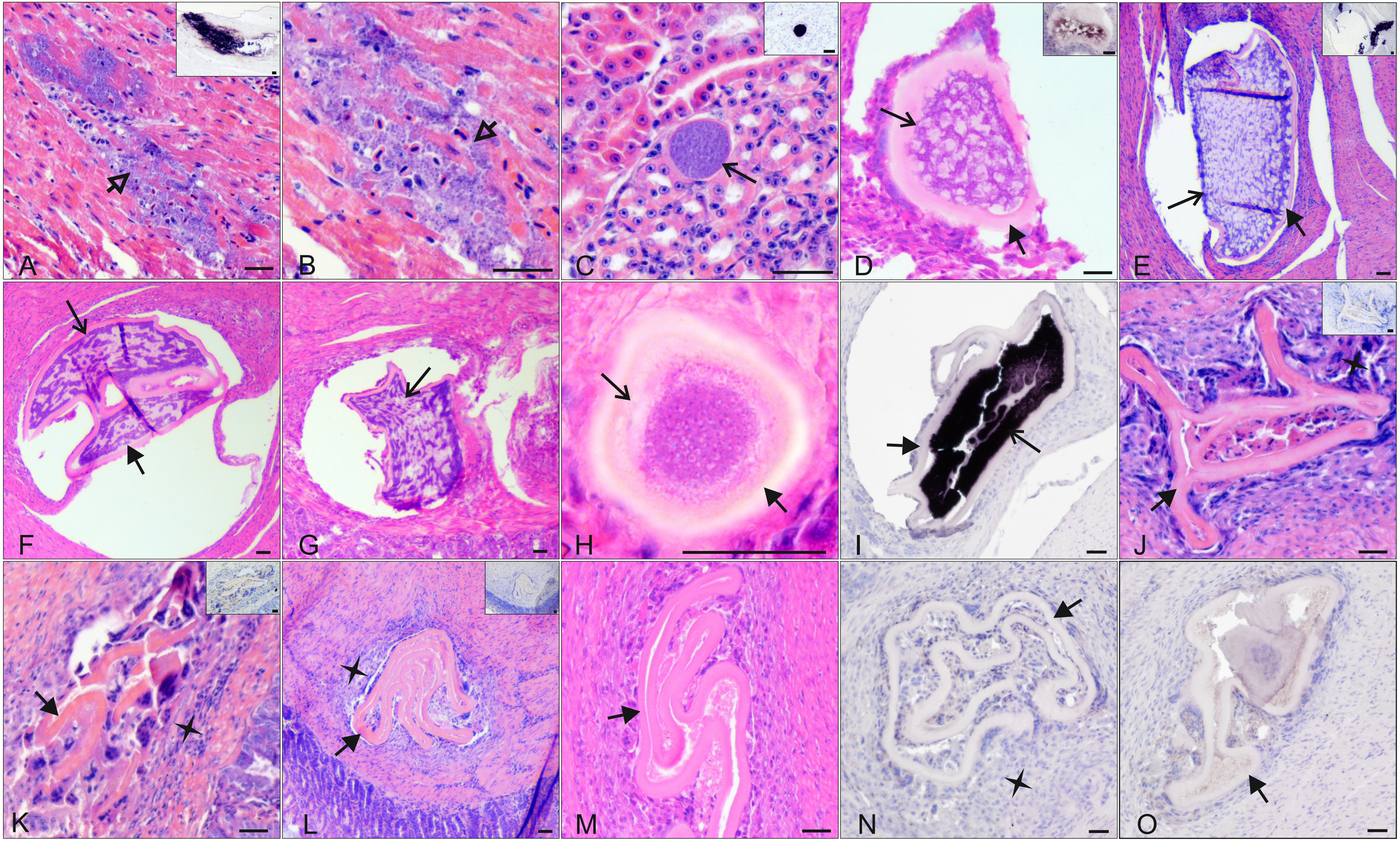

Figure S4. Megalomeronts (C–O) and merozoites (A–B) of *Haemoproteus majoris* lineage hWW2 in *Sylvia atricapilla* '24 Sp' in hematoxylin and eosin (H&E) stained-sections and their corresponding images after chromogenic *in situ* hybridization (CISH) treatment (inserts and I, N, O): in heart (A, B), kidneys (C), and gizzard (D–O). Note the more densely located and connected cytomeres in developing megalomeronts (D–G). Young megalomeronts (IC, H) and ruptured megalomeronts (J–O) were observed. Inflammatory reactions were observed around several megalomeronts (J–L, N). Megalomeronts were surrounded by a thick capsular-like wall. Cytomeres were readily visible. Free merozoites were found scattered between muscle fibers in one place of the heart (A, B). Long arrow: megalomeront; short arrow: capsular-like wall; opened arrow: merozoites; cross: inflammatory reaction. Scale bar: 25 μm.
