# Supplementary material for: Comparative Analysis of the Exo-Erythrocytic Development of Five Lineages of Haemoproteus majoris, a Common Haemosporidian Parasite of European Passeriform Birds
Source: Pathogens. 2023 Jun 30;12(7):898. doi: 10.3390/pathogens12070898 (PMC10386383; doi:10.3390/pathogens12070898)
Supplement: Supplementary file 1 [file pathogens-12-00898-s001.zip › Figure S5.pdf]

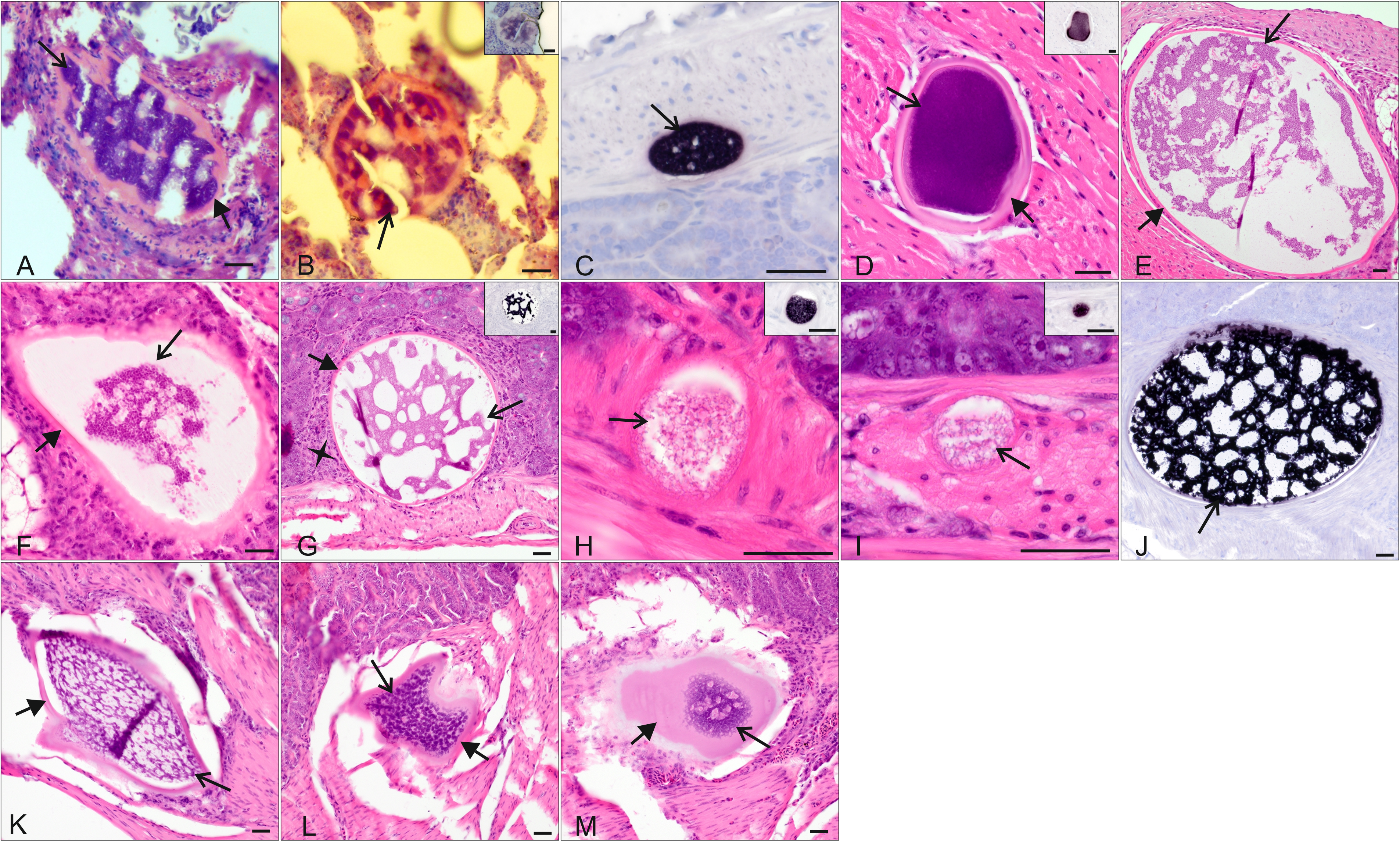

Figure S5. Megalomeronts of *Haemoproteus majoris* found in co-infections with several of its lineages in hematoxylin and eosin (H&E) stained-sections and their corresponding images after chromogenic *in situ* hybridization (CISH) treatment (inserts and C, J): in *Parus major* '4 Sp' (co–infection of hWW2/hCWT4/hPARUS1/hPHSIB1 lineages) liver (A–B), *Phylloscopus sibilatrix* '17 Sp' (co-infection of hWW2 and hPHSIB1 lineages) intestine (C), *Phoenicurus ochruros* '20 Sp' (co-infection of hPHSIB1 and hWW2 lineages) heart (D–F), intestine (G–J) and gizzard (K–M). Note the variously shaped interconnected cytomeres in developing megalomeronts (E–G) and the more densely located and connected cytomeres in advanced developing megalomeronts (J–L). Very young megalomeronts (H, I) and young megalomeronts (C, D) were observed in H&E and CISH sections. Megalomeronts were found alone in the tissues and sometimes several were found very close to each other in the same section (K, AD). Inflammatory reactions were observed around several of the megalomeronts (E, H, J, K, AP). Megalomeronts were surrounded by a thick capsular-like wall. Cytomeres were readily visible. Long arrow: megalomeront; short arrow: capsular-like wall; cross: inflammatory reaction; arrowhead: enlarged host cell nucleus. Scale bar: 25 μm.
